# Salusin-*β* contributes to oxidative stress and inflammation in diabetic cardiomyopathy

**DOI:** 10.1038/cddis.2017.106

**Published:** 2017-03-23

**Authors:** Ming-Xia Zhao, Bing Zhou, Li Ling, Xiao-Qing Xiong, Feng Zhang, Qi Chen, Yue-Hua Li, Yu-Ming Kang, Guo-Qing Zhu

**Affiliations:** 1 Department of Physiology, Key Laboratory of Cardiovascular Disease and Molecular Intervention, Nanjing Medical University, Nanjing, China; 2 Department of Pathophysiology, Nanjing Medical University, Nanjing, China; 3 Department of Physiology and Pathophysiology, Cardiovascular Research Center, Xi'an Jiaotong University School of Medicine, Xi'an, China

## Abstract

Salusin-*β* accelerates inflammatory responses in vascular endothelial cells, and increases oxidative stress in vascular smooth muscle cells. Plasma salusin-*β* levels were increased in diabetic patients. This study was designed to determine whether salusin-*β* is involved in the pathogenesis of diabetic cardiomyopathy (DCM), and whether knockdown of salusin-*β* attenuates cardiac inflammation and oxidative stress in rats with DCM. H9c2 or neonatal rat cardiomyocytes were incubated with 33.3 mM of glucose to mimic the high glucose (HG) in diabetes. Streptozotocin and high-fat diet were used to induce type 2 diabetes in rats. HG induced salusin-*β* expression in H9c2 cells. Salusin-*β* caused greater responses of oxidative stress, NF*κ*B activation and inflammation in HG-treated H9c2 cells than these in control H9c2 cells. Diphenyleneiodonium (a NAD(P)H oxidase inhibitor) or *N*-acetylcysteine (an antioxidant) inhibited the salusin-*β*-induced NF*κ*B activation and inflammation. Bay11-7082 (a NF*κ*B inhibitor) attenuated salusin-*β*-induced inflammation but not oxidative stress. Knockdown of salusin-*β* prevented the HG-induced oxidative stress, NF*κ*B activation and inflammation in neonatal rat cardiomyocytes. Silencing salusin-*β* with adenoviruse-mediated shRNA had no significant effects on blood glucose and insulin resistance, but attenuated ventricular dysfunction in diabetic rats. Oxidative stress, NF*κ*B activation, inflammation, salusin-*β* upregulation in myocardium of diabetic rats were prevented by knockdown of salusin-*β*. These results indicate that salusin-*β* contributes to inflammation in DCM via NOX2/ROS/NF*κ*B signaling, and that knockdown of salusin-*β* attenuates cardiac dysfunction, oxidative stress and inflammation in DCM.

Diabetic cardiomyopathy (DCM) comprises structural and functional abnormalities of the myocardium in diabetic patients or animals without hypertension or coronary artery disease.^1, 2^ In diabetic heart, metabolic derangements, impairments in excitation–contraction coupling, loss of normal microvessels and remodeling of the extracellular matrix are involved in contractile dysfunction.^3^ It is known that diabetes or hyperglycemia increases intracellular reactive oxygen species (ROS), which subsequently induces injury and inflammation.^4^ Oxidative stress is closely associated with the pathogenesis of diabetes, and long-term exposure to oxidative stress in diabetes induces chronic inflammation.^5^ Oxidative stress may have a critical role in the development of DCM.^6^

Salusin-*β* is identified as a bioactive peptide of hemodynamic and mitogenic activities.^7^ The initial 18 amino acids of human salusin-*β* have high homology with the N-terminal sequence of rat salusin.^8^ Previous studies in our lab have shown that central salusin-*β* contributes to sympathetic activation, arginine vasopressin release and hypertension.^9, 10, 11^ Salusin-*β* promotes proliferation of vascular smooth muscle cells (VSMCs) and vascular fibrosis.^12^ ROS production in VSMCs mediates salusin-*β*-induced foam cell formation and monocyte adhesion,^13^ VSMCs migration and intimal hyperplasia after vascular injury.^14^ Knockdown of salusin-*β* reduces ROS production in injured carotid arteries in rats.^13^ Salusin-*β* has been implicated in inflammatory response in vascular endothelial cells.^15, 16^ Patients with diabetes displayed a distinctly increase in plasma salusin-*β* levels.^17^ Salusin-*β* is widely distributed in a host of tissues,^8^ and can be synthesized locally in the muscle cells of the heart.^18^ It has been found that salusin-*β* contributes to the development of coronary ligation-induced myocardial infarction in rats, and inhibition of endogenous salusin-*β* may be useful to suppress ventricular remodeling after myocardial ischemia.^19^ We hypothesized that salusin-*β* may have a critical role in DCM. In this study, we sought to explore the roles of salusin-*β* in type 2 DCM and the underlying molecular mechanism. This study was designed to determine whether salusin-*β* was involved in the oxidative stress and inflammation of DCM. Furthermore, the effects of knockdown of salusin-*β* on cardiac oxidative stress and inflammation were investigated in rats with DCM.

## Results

### Salusin-*β* induces inflammation and oxidative stress in cardiomyocytes

**Figure 1:**
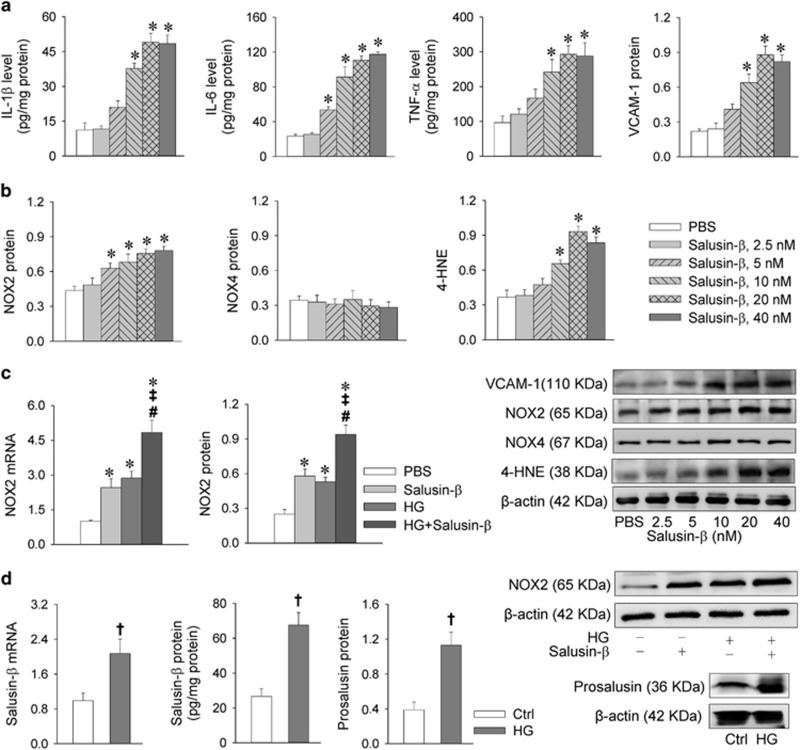
Effects of salusin-*β* on inflammation and oxidative stress and effects of HG on salusin-*β* and NOX2 expression levels in H9c2 cells. (**a**) Effects of salusin-*β* on inflammation in H9c2 cells. (**b**) Effects of salusin-*β* on oxidative stress in H9c2 cells. H9c2 cells were treated with different doses of salusin-*β* (0, 2.5, 5, 10, 20 or 40 nM) for 24 h. ELISA was used to measure the levels of salusin-*β*, IL-1*β*, IL-6 and TNF-*α*. Western blotting was used to detect the relative levels VCAM-1, NOX2, NOX4 and 4-HNE. (**c**) Effects of salusin-*β* (20 nM for 24 h) and HG (33.3 mM for 24 h) on NOX2 mRNA and NOX2 expression levels. (**d**) Salusin-*β* mRNA, salusin-*β* protein and prosalusin levels in H9C2 cells treated with HG (33.3 mM) for 24 h. Values are mean±S.E.M. *P<0.05 *versus* PBS, ^†^*P*<0.05 *versus* Ctrl. ^‡^*P*<0.05 *versus* Salusin-*β*. ^#^*P*<0.05 *versus* HG. *n*=6

Salusin-*β* increased the levels of pro-inflammatory cytokines including interleukin-1*β* (IL-1*β*), IL-6 and tumor necrosis factor-*α* (TNF-*α*) in H9c2 cells. TNF-*α* is known to aggravate heart inflammation via the upregulation of vascular cell adhesion molecule-1 (VCAM-1).^20^ 4-Hydroxynonenal (4-HNE) is a major marker of oxidative stress.^21, 22^ Salusin-*β* increased VCAM-1, NOX2 and 4-HNE expression levels, but had no significant effect on NOX4 expression in H9c2 cells (Figures 1a and b). These results indicate that salusin-*β* causes inflammation and oxidative stress in H9c2 cells. High glucose (HG) caused similar increases in NOX2 mRNA and protein expression levels in H9c2 cells, and combined administration of HG and salusin-*β* caused greater effects than salusin-*β* or HG alone (Figure 1c). However, insulin had no significant effects on the salusin-*β* and NOX2 expression levels in both baseline or HG state in H9c2 cells (Supplementary Figure 1).

### HG increases salusin-*β* expression in cardiomyocytes

It is known that plasma salusin-*β* levels are increased in diabetic patients.^17^ Treatment with HG for 24 h not only increased salusin-*β* mRNA levels, but also upregulated salusin-*β* and its precursor prosalusin protein expression levels in H9c2 cells (Figure 1d), suggesting that HG is a stimulator of salusin-*β* expression.

### ROS contributes to HG- and salusin-*β*-induced inflammation and NF*κ*B activation in cardiomyocytes

**Figure 2:**
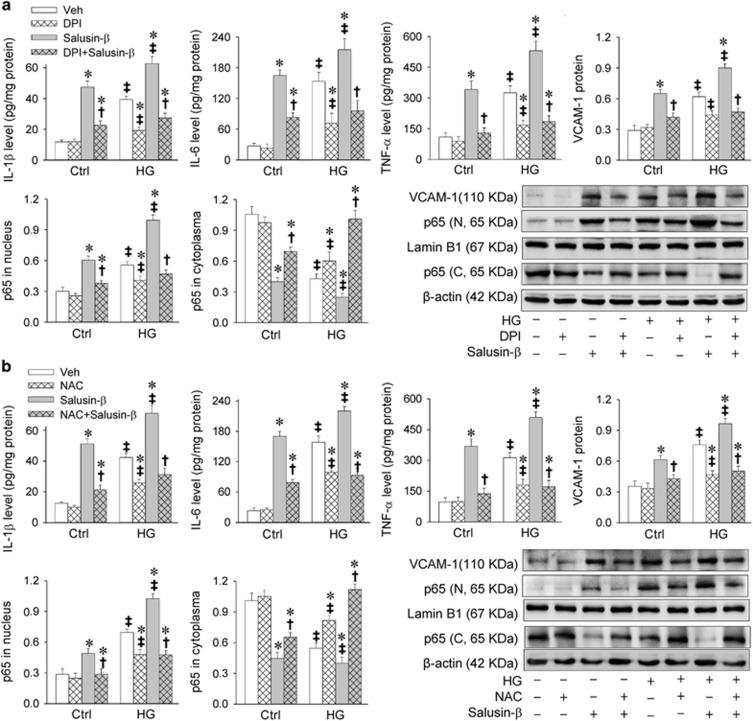
Effects of DPI (a NAD(P)H oxidase inhibitor) and NAC (an antioxidant) on salusin-*β*-induced inflammation and NF*κ*B activation in H9c2 cells. H9c2 cells were pretreated with DPI (10 *μ*M) or NAC (5 mM) for 1 h before incubated with salusin-*β* (20 nM) or both salusin-*β* (20 nM) and HG (33.3 mM) for 24 h. (**a**) Effects of DPI on salusin-*β*-induced inflammation and NF*κ*B activation. (**b**) Effects of NAC on salusin-*β*-induced inflammation and NF*κ*B activation. Values are mean±S.E.M. *P<0.05 *versus* Veh; ^†^*P*<0.05 *versus* Salusin-*β*. ^‡^*P*<0.05 *versus* Ctrl. *n*=6

HG is known to activate NF*κ*B.^23, 24^ In this study, HG caused inflammation evidenced by the upregulation of IL-1*β*, IL-6, TNF-*α* and VCAM-1, and NF*κ*B activation indicated by the increased p65 in nucleus and reduced p65 in cytoplasm in H9c2 cells. Salusin-*β* induced greater responses in inflammation and NF*κ*B activation in HG-treated H9c2 cells than those in control cells. Inhibition of NAD(P)H oxidase (NOX) with diphenyleneiodonium (DPI) not only attenuated the HG-induced inflammation and NF*κ*B activation, but also salusin-*β*-induced inflammation and NF*κ*B activation in both HG-treated H9c2 cells and control cells (Figure 2a). Antioxidant *N*-acetylcysteine (NAC) showed similar effects with DPI in attenuating HG- and salusin-*β*-induced inflammation and NF*κ*B activation (Figure 2b).

### Inhibition of NF*κ*B attenuates HG- and salusin-*β*-induced inflammation in cardiomyocytes

**Figure 3:**
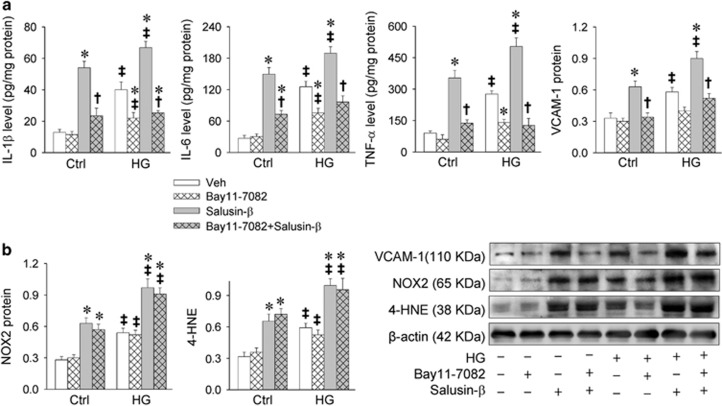
Effects of Bay11-7082 (a NF*κ*B inhibitor) on salusin-*β*-induced inflammation and oxidative stress in H9c2 cells. H9c2 cells were pretreated with Bay11-7082 (10 *μ*M) for 1 h before incubated with salusin-*β* (20 nM) or both salusin-*β* (20 nM) and HG (33.3 mM) for 24 h. (**a**) Effects of Bay11-7082 on salusin-*β*-induced inflammation. (**b**) Effects of Bay11-7082 on salusin-*β*-induced oxidative stress. Values are mean±S.E.M. *P<0.05 *versus* Veh; ^†^*P*<0.05 *versus* Salusin-*β*. ^‡^*P*<0.05 *versus* Ctrl. *n*=6

Inhibition of NF*κ*B with Bay11-7082 attenuated salusin-*β*- or HG-induced inflammation in H9c2 cells (Figure 3a). However, Bay11-7082 had no significant effects on HG- or salusin-*β*-induced NOX2 and 4-HNE expression levels (Figure 3b).

### Knockdown of salusin-*β* attenuates HG-induced inflammation and oxidative stress in cardiomyocytes

**Figure 4:**
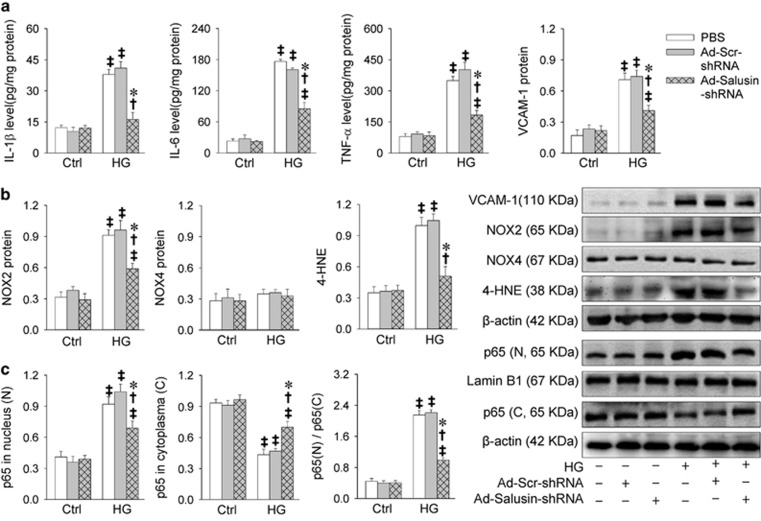
Effects of knockdown of salusin-*β* with adenoviral vectors encoding salusin-*β* shRNA (Ad-Salusin-shRNA) on inflammation, oxidative stress and NF*κ*B activation in neonatal rat cardiomyocytes. The cardiomyocytes were treated with both Ad-Salusin-shRNA (MOI=100) and HG (33.3 mM) for 24 h. Adenoviral vectors encoding scramble shRNA (Ad-Scr-shRNA) was used as a control of Ad-salusin-shRNA. (**a**) Effects of Ad-Salusin-shRNA on inflammation. (**b**) Effects of Ad-Salusin-shRNA on oxidative stress. (**c**) Effects of Ad-Salusin-shRNA on NF*κ*B activation. Values are mean±S.E.M. *P<0.05 *versus* PBS; ^†^*P*<0.05 *versus* Ad-Scr-shRNA. ^‡^*P*<0.05 *versus* Ctrl. *n*=6

Knockdown of salusin-*β* with Ad-Salusin-shRNA dose- and time-relatedly reduced salusin-*β*, IL-1*β*, IL-6 and TNF-*α* mRNA levels, and NOX2 protein and 4-HNE levels in HG-treated H9c2 cells, reaching its maximal effects at the multiplicity of infection (MOI)=100 (Supplementary Figure 2) treated for 24 h (Supplementary Figure 3). To confirm these findings, we further examined the effects of salusin-*β*-shRNA in neonatal rat cardiomyocytes. We found that salusin-*β*-shRNA attenuated HG-induced inflammation, NOX2 expression, ROS production and NF*κ*B activation in HG-treated neonatal rat cardiomyocytes, but had no significant effects in control cells (Figures 4a–c).

### Salusin-*β* knockdown improves ventricle functions in diabetic rats

**Figure 5:**
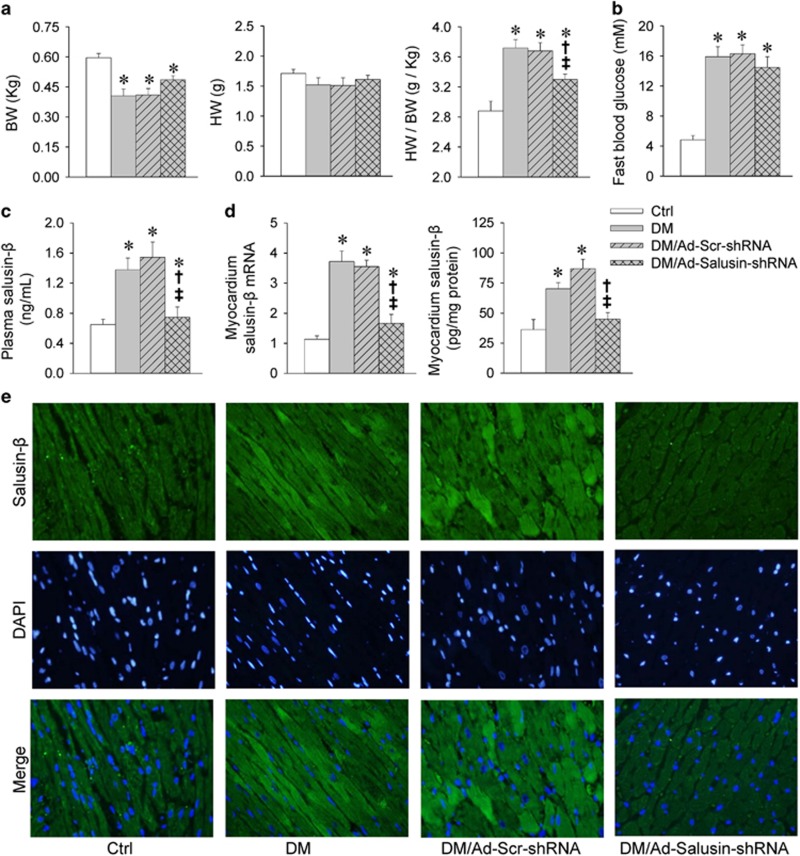
Effects of interrupting salusin-*β* on body weight (BW), heart weight (HW), fast blood glucose and salusin-*β* expression levels in rats with diabetes (DM). Intravenous injection of adenoviral vectors encoding salusin-*β* shRNA (Ad-Salusin-shRNA, 2.0 × 10^10^ plaque-forming units) or scramble shRNA (Ad-Scr-shRNA) were carried out and repeated for 2 weeks in rats. The measurements were made 4 weeks after the first adenovirus transfer. (**a**) BW, HW and HW/BW. (**b**) Fast blood glucose. (**c**) Plasma salusin-*β* levels. (**d**) Salusin-*β* mRNA and protein expression in myocardium. (**e**) Immunofluorescence staining for salusin-*β* in myocardium. Values are mean±S.E.M. *P<0.05 *versus* Ctrl; ^†^*P*<0.05 *versus* DM; ^‡^*P*<0.05 *versus* DM/Ad-Scr-shRNA. *n*=6

Ratio of heart weight to body weight was increased in rats with diabetes mellitus (DM), which was attenuated by Ad-Salusin-shRNA (Figure 5a). Echocardiographic assessment showed that left ventricular internal dimensions (LVIDs), left ventricular mass (LVM)/BW were increased, whereas ejection fraction (EF) and fractional shortening (FS) were reduced in diabetic rats, which were attenuated by Ad-Salusin-shRNA (Table 1). These results indicate that knockdown of salusin-*β* improves cardiomyopathy in type 2 diabetes.

### Salusin-*β* knockdown fails to attenuate hyperglycemia and insulin resistance in diabetic rats

Diabetic rats showed much higher fast blood glucose level than that in control rats, which was not affected by Ad-Salusin-shRNA (Figure 5b). Insulin tolerance test (ITT) was used for evaluating insulin resistance, and the efficiency of insulin was quantified by its ability in reducing blood glucose level. Insulin was less effective in DM rats than that in control rats, suggesting impaired insulin sensitivity in diabetic rats. Ad-Salusin-shRNA had no significant role in improving the insulin sensitivity in diabetic rats. Glucose tolerance test (GTT) was used for evaluating glucose tolerance. Diabetic rats manifested significantly elevated glucose excursions following glucose challenge compared with control rats. Ad-Salusin-shRNA had no significant effect on the glucose excursion (Supplementary Figure 4).

### Salusin-*β* expression levels in diabetic rats

Plasma salusin-*β* levels were increased in diabetic rats. Salusin-*β* mRNA and protein expression levels in myocardium were unregulated in diabetic rats. Furthermore, immunofluorescence staining showed the enhanced salusin-*β* immunofluorescence in myocardium of diabetic rats. Ad-Salusin-shRNA significantly reduced plasma salusin-*β* levels and salusin-*β* expression in myocardium of diabetic rats (Figures 5c–e).

### Salusin-*β* knockdown attenuates inflammation in diabetic rats

**Figure 6:**
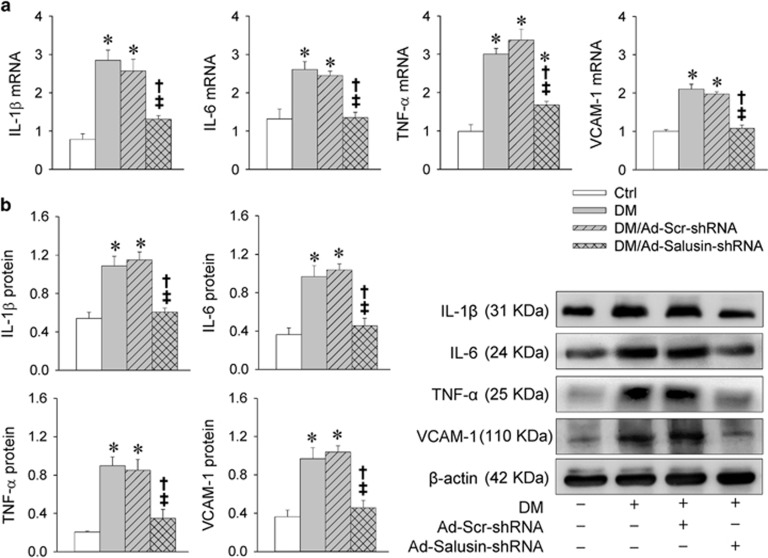
Effects of interrupting salusin-*β* on inflammation in myocardium in rats with diabetes (DM). Intravenous injection of adenoviral vectors encoding salusin-*β* shRNA (Ad-Salusin-shRNA, 2.0 × 10^10^ plaque-forming units) or scramble shRNA (Ad-Scr-shRNA) were carried out and repeated for 2 weeks in rats. The measurements were made 4 weeks after the first adenovirus transfer. (**a**) Relative mRNA levels of IL-1*β*, IL-6, TNF-*α* and VCAM-1. (**b**) Relative protein expression levels of IL-1*β*, IL-6, TNF-*α* and VCAM-1. Values are mean±S.E.M. *P<0.05 *versus* Ctrl; ^†^*P*<0.05 *versus* DM; ^‡^*P*<0.05 *versus* DM/Ad-Scr-shRNA. *n*=6

IL-1*β*, IL-6, TNF-*α* and VCAM-1 mRNA levels were increased in diabetic rats, which were prevented by salusin-*β* knockdown with Ad-Salusin-shRNA (Figure 6a). Similarly, IL-1*β*, IL-6, TNF-*α* and VCAM-1 proteins were upregulated in diabetic rats, which were attenuated by Ad-Salusin-shRNA (Figure 6b).

### Salusin-*β* knockdown reduces NF*κ*B activation, NOX2 expression and ROS production in diabetic rats

**Figure 7:**
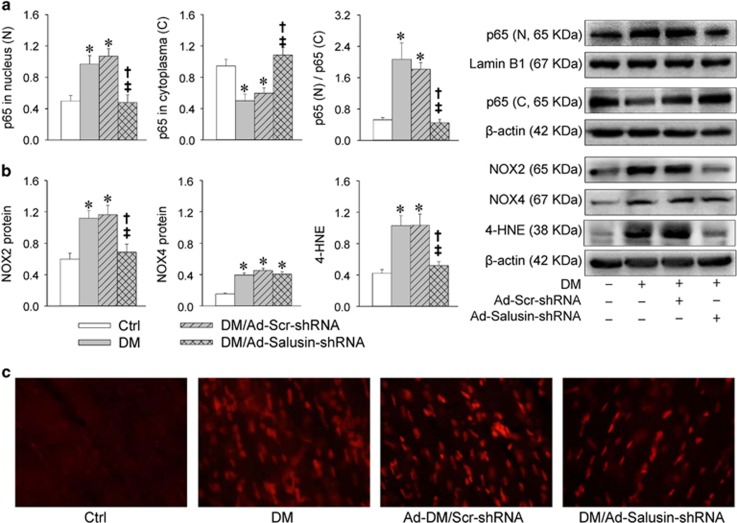
Effects of interrupting salusin-*β* on NF*κ*B activation and oxidative stress in myocardium in rats with diabetes (DM). Intravenous injection of adenoviral vectors encoding salusin-*β* shRNA (Ad-Salusin-shRNA, 2.0 × 10^10^ plaque-forming units) or scramble shRNA (Ad-Scr-shRNA) were carried out and repeated for 2 weeks in rats. The measurements were made 4 weeks after the first adenovirus transfer. (**a**) NF*κ*B activation. (**b**) Oxidative stress. (**c**) DHE staining for detecting ROS in myocardium. Values are mean±S.E.M. *P<0.05 *versus* Ctrl; ^†^*P*<0.05 *versus* DM; ^‡^*P*<0.05 *versus* DM/Ad-Scr-shRNA. *n*=6

Diabetic rats showed increased p65 in nucleus and reduced p65 in cytoplasm, which were prevented by Ad-Salusin-shRNA (Figure 7a). NOX2 and NOX4 expression levels, as well as 4-HNE levels, were increased in myocardium of diabetic rats. Ad-Salusin-shRNA reduced NOX2 expression levels and 4-HNE levels, but not NOX4 expression in diabetic rats (Figure 7b). Dihydroethidium (DHE) fluorescent dye further confirmed that increased ROS production in diabetic myocardium were attenuated by Ad-Salusin-shRNA (Figure 7c).

## Discussion

Sustained increased inflammatory cytokines in myocardium are involved in augmenting remodeling process.^25^ The levels of inflammatory cytokines in myocardium were increased in diabetic animal models.^26^ Accumulating evidences suggest increased oxidative stress coupled with activation of downstream pro-inflammatory cytokines have pivotal roles in the development of DCM.^26, 27, 28, 29^ It has been found that plasma salusin-*β* levels were increased in patients with diabetes.^17^ ROS production was involved in the effects of salusin-*β* in brain^10^ and VSMCs.^14^ The primary novel findings in this study are that salusin-*β* contributes to oxidative stress and inflammation in DCM via NF*κ*B signaling, and that knockdown of salusin-*β* attenuates cardiac dysfunction, oxidative stress and inflammation in DCM.

Cardiomyocytes cultured in HG was used as a model to mimic the increased glucose level in diabetes.^30^ HG increased salusin-*β* mRNA levels and upregulated salusin-*β* and its precursor prosalusin protein expression levels in H9c2 cells, suggesting that HG is a stimulator of salusin-*β* expression at both transcriptional and post-translational levels. Either HG or salusin-*β* alone caused inflammation, NOX2 upregulation and oxidative stress in H9c2 cells, which were further confirmed in neonatal rat cardiomyocytes. HG or salusin-*β* increased NOX2 mRNA levels, suggesting transcriptional regulation is involved in the HG- or salusin-*β*-induced NOX2 upregulation. As diabetic patients showed an increase in plasma salusin-*β* levels,^17^ the effects of combined salusin-*β* and HG were examined *in vitro* to mimic the upregulated salusin-*β* and hyperglycemia situations of diabetes. Salusin-*β* induced more potent inflammation, NOX2 upregulation and oxidative stress responses in HG-treated H9c2 cells than those in control cells. Knockdown of salusin-*β* with shRNA in neonatal rat cardiomyocytes attenuated HG-induced inflammation and NOX2 upregulation and oxidative stress. Inhibiting NOX with DPI or scavenging ROS with NAC attenuated inflammation in both control and HG-treated H9c2 cells. These results indicate that salusin-*β* is a stimulator of inflammation and oxidative stress in cardiomyocytes, and salusin-*β* is at least partially responsible for HG-induced inflammation mediated by NOX2-derived ROS production.

NF*κ*B is a transcription factor that is activated by various stimuli such as cytokines, ROS, bacterial or viral products. Activated NF*κ*B translocates into the nucleus and stimulates related gene expression.^31^ As a key transcription factor related to inflammation, NF*κ*B has been shown to have a pivotal role in the development of DCM.^32^ NF*κ*B activation has been found in primary human cardiomyocytes exposed to HG.^26^ In this study, HG-induced NF*κ*B activation in both H9c2 and neonatal rat cardiomyocytes. Salusin-*β* caused greater NF*κ*B activation response in HG-treated H9c2 cells than those in control cells. Knockdown of salusin-*β* with shRNA in neonatal rat cardiomyocytes inhibited HG-induced NF*κ*B activation. Inhibiting NOX with DPI or scavenging ROS with NAC attenuated salusin-*β*-induced NF*κ*B activation in both control and HG-treated H9c2 cells. Inhibition of NF*κ*B with Bay11-7082 had no significant effects on salusin-*β*-induced NOX upregulation and ROS production, but attenuated salusin-*β*-induced inflammation in both control and HG-treated H9c2 cells. These results indicate that NF*κ*B pathway mediates HG- or salusin-*β*-induced inflammation via NOX2-derived ROS production in cardiomyocytes.

Low-dose streptozotocin (STZ) combined with high-fat diet (HFD)-induced diabetes in rodents has been identified as an ideal animal model of type 2 diabetes, which simulates the metabolic characteristics and disease progression of type 2 diabetes.^33, 34^ This model is appropriate for testing anti-diabetic agents in treatment of type 2 diabetes.^35^ In this study, combined treatment with STZ and HFD caused increases in fasting blood glucose, insulin resistance and ratio of heart weight to body weight, and decreases in glucose tolerance and ventricular function in rats. These results confirm that the type 2 diabetes with cardiomyopathy rat model was successfully established, which is consistently with previous studies.^36, 37, 38^ NOX2 upregulation, oxidative stress, NF*κ*B activation and inflammation were found in the myocardium of DM rats, which were attenuated by silencing salusin-*β* gene in diabetic rats. Furthermore, plasma salusin-*β* levels and myocardium salusin-*β* expression levels in myocardium were increased in diabetic rats. More importantly, silencing salusin-*β* gene improved ventricular dysfunction in DM. These results indicate the important role of salusin-*β* in the pathogenesis of DCM, and further support the findings that HG stimulates salusin-*β* expression, which causes oxidative stress followed by NF*κ*B activation and inflammation. Salusin-*β* may be taken as a target in attenuating cardiomyopathy in DM.

Insulin had no significant effects on the baseline salusin-*β* and NOX2 expression levels or on the HG-induced upregulation of salusin-*β* and NOX2 in H9c2 cells, suggest that insulin is not involved in the HG-induced salusin-*β* and NOX2 expression levels *in vitro*. Knockdown of salusin-*β* had no significant effects in attenuating hyperglycemia and insulin resistance in DM rats, which suggests that salusin-*β* is not involved in the pathogenesis of hyperglycemia and insulin resistance in diabetes. The beneficial roles of knockdown of salusin-*β* in attenuating oxidative stress and inflammation in diabetes are independently of blood glucose and insulin resistance in DM rats.

In conclusion, salusin-*β* promotes cardiac inflammation via NOX2-derived ROS production and nucleus translocation of p65-NF*κ*B. Knockdown of salusin-*β* attenuates cardiac dysfunction, oxidative stress and inflammation in DCM. Our findings provided a new insight toward the development of therapeutic agents aimed at reducing cardiac inflammation and oxidative stress in DCM.

## Materials and methods

All experiments adhered to the Care and Use of Laboratory Animal published by the US National Institutes of Health (NIH publication, 8th edition, 2011) and approved by the Experimental Animal Care and Use Committee of Nanjing Medical University.

### Cell culture of H9c2 cells and neonatal rat cardiomyocytes

H9c2 rat cardiomyoblast cells were obtained from Costar Corning Inc. (Corning, CA, USA). Neonatal rat cardiomyocytes were isolated enzymatically from 1 to 3 days old SD rats (Animal Center of Nanjing Medical University, Nanjing, China). Briefly, left ventricles were minced and digested with 0.04% collagenase II (Sigma, St. Louis, MO, USA). The supernatant-containing suspended cells were preplated for 1.5 h to remove non-myocytes. Then, the cardiomyocytes were seeded onto culture plates at about 5 × 10^4^ cells/cm^2^, and cultured in the medium containing DMEM/F-12 with HEPES (Hyclone, Beijing, China), 10% FBS (Gibco, Grand Island, NY, USA), 1% penicillin and 1% streptomycin (Solarbio, Beijing, China) with 5% CO_2_ at 37 °C. Cardiomyocytes were seeded for 3 days before use. In order to mimic the increased glucose level in diabetes, the H9c2 cells or primary neonatal cardiomyocytes were cultured in 33.3 mM glucose (HG) or 5.5 mM glucose (normal glucose), the latter of which was used as a control for indicated time.^30^

### Rat model of type 2 diabetes and knockdown of salusin-*β*

Combination of low-dose STZ and HFD were used to induce type 2 diabetes.^33, 34, 35^ Control diet (20% protein, 3% fat, 3% fiber and 74% carbohydrate) and HFD (34.5% fat, 17.5% protein and 48% carbohydrate) were obtained from Beijing HFK Bio-Technology Co. Ltd (Beijing, China). Male SD rats weighing 120–140 g were used in the experiments, which were housed in a temperature-controlled room with 12-h light–dark cycles, and had free access to standard chow and tap water. After 2 weeks of acclimatization, rats were randomized divided into four groups. One group of rats (Ctrl group) received an intraperitoneal injection of vehicle or PBS, and were fed with control diet throughout the experiment. Another three groups of rats combined into one group to induce type 2 diabetes. These rats were fed with HFD instead of previously used control diet during the following experiments. After 4 weeks HFD feeding, the diabetic rats were subjected to 12-h fasting followed by intraperitoneal injection of low-dose of STZ (27.5 mg/kg body weight, dissolved in 0.1 M citrate buffer, pH 4.5).^36, 38^ Twelve weeks after injection of STZ (16 weeks HFD), diabetes was confirmed by the fast blood glucose >11.1 mmol/l.^30, 38^ The diabetic rats were re-divided into three groups, which respectively received intravenous injection of PBS (DM group), adenoviral vectors encoding scramble shRNA (2.0 × 10^10^ plaque-forming units, DM/Ad-Scr-shRNA group) or adenoviral vectors encoding salusin-*β* shRNA (2.0 × 10^10^ plaque-forming units, DM/Ad-Salusin-shRNA group). The intravenous injections were repeated 2 weeks after the first administration.^14, 38^ The rats were killed for measurements 4 weeks after the first time of intravenous injection (20 weeks after feeding with HFD). Ad-Salusin-shRNA and Ad-Scr-shRNA were constructed by Genomeditech Co. (Shanghai, China), which downregulated the salusin-*β* expression by 75%. The efficiency of Ad-Sal-shRNA in knockdown of salusin-*β* was confirmed in rats in our previous study.^14^ The sequences of salusin-*β*-shRNA are listed in a table (Supplementary Table 1). The experimental protocols were summarized in a schematic diagram (Supplementary Figure 5).

### ITT and GTT

Rats were fasted for 4 h before intraperitoneal injection of insulin (1 units/kg body weight) for ITT, and fasted for 12 h before glucose (1 g/kg body weight) for GTT. Blood glucose in tail veins were measured with a blood glucometer (One Touch, Milpitas, CA, USA) at 0, 15, 30, 60 and 120 min after the injection.^38^

### Echocardiography

Cardiac function was measured with Vevo2100 imaging system (VisualSonics, Toronto, Canada). All measurements were the average of six consecutive cardiac cycles and performed by the same operator. The derived echocardiography parameters included LVID, interventricular septum thickness, left ventricular posterior wall dimension, left ventricular volume, LVM, EF and FS.

### ELISA

Commercial ELISA kits (Uscn Life Science, Houston, TX, USA) were used for the measurement of salusin-*β*, IL-1*β*, IL-6 and TNF-*α* in according to the manufacturer's instructions. Briefly, the samples were collected and added into the pre-coated ELISA plate (100 *μ*l per well). Plates were incubated at 37 °C for 2 h, washed and then incubated with conjugated solution for 1 h at 37 °C. Finally, the reactions were stopped with stop solution, and optical density was determined by use of a microplate reader (ELX800, BioTek, Winoosk, VT, USA) at 450 nm.

### Western blot analysis

Prosalusin, VCMA-1, NOX2, NOX4, 4-HNE, p65, IL-1*β*, IL-6 and TNF-*α* were determined with western blot analysis. Briefly, tissues or cardiomyocytes were sonicated in RIPA lysis buffer and homogenized. The debris was removed, and the supernatant was obtained by centrifugation at 4 °C. Total or nuclear proteins were extracted using commercially available kits (Beyotime Biotechnology, Shanghai, China) according to the manufacturer's protocol. Equal amounts of protein were separated on sodium dodecyl sulfate-polyacrylamide gel electrophoresis and transferred onto PVDF membrane. Blocking was made at room temperature with 5% nonfat milk powder prepared in Tris-buffered saline containing 0.1% Tween 20. Then, membranes were incubated overnight at 4 °C with the primary antibodies followed by incubation with appropriate HRP-linked secondary antibody. The protein expression levels were visualized by enhanced chemiluminescence (Millipore, Billerica, MA, USA). Primary antibodies of IL-6, TNF-*α*, NOX2, NOX4, 4-HNE and secondary antibodies were purchased from Abcam (Cambridge, MA, USA). Antibodies of VCMA-1, IL-1*β*, p65 and *β*-actin were obtained from Biotechnology Inc. (Dallas, TX, USA). Antibodies of lamin B1 was purchased from Santa Cruz Biotechnology Co. (Santa Cruz, CA, USA). Antibodies of prosalusin was obtained from Proteintech Group Inc. (Rosemont, IL, USA).

### RT-PCR

Salusin-*β*, NOX2, IL-1*β*, IL-6, TNF-*α* and VCAM-1 mRNA were analyzed by real-time quantitative PCR. Total RNA was separated using a Trizol reagent (Life Technologies, Gaithersburg, MD, USA) according to the manufacturer's protocols. RNA concentrations and purity were assessed by the measurement of optical density at 260 and 280 nm. Reverse transcriptase reactions were made using the PrimeScript RT reagent Kits (Takara, Otsu, Shiga, Japan) according to the manufacturer's instruction. Real-time PCR was performed with Quantitative PCR with SYBR Premix Ex Taq TM (Takara), and ABI PRISM 7500 sequence detection PCR system (Applied Biosystems, Foster City, CA, USA). The samples were relatively quantified by normalizing the targeted gene level to that of internal control by the ΔΔCt method. The sequences of primers were listed in Supplementary Table 2.

### DHE fluorescence staining

ROS production in myocardium was evaluated with DHE staining. Left ventricles were excised from rats, and immediately embedded in OCT compound. The tissues were cut into 25 *μ*m thick sections, and then were incubated with DHE (10 *μ*mol/l) in PBS in a dark and humidified container at 37 °C for 5 min. DHE is oxidized upon reaction with superoxide to ethidium bromide, which binds to DNA in the nucleus. The fluorescence were viewed under the fluorescence microscope (DP70, Olympus Optical, Tokyo, Japan).

### Salusin-*β* immunofluorescence

Paraffin-embedded sections were permeabilized using 0.1% Triton X-100 for 10 min after deparaffinization and rehydration. The sections were washed in PBS then blocked with 10% goat serum for 1 h, and then incubated with rabbit anti-salusin-*β* antibody overnight at 4 °C. The secondary TRITC-conjugated goat anti-rabbit IgG (1 : 400) was used. Nuclei were stained with 4′,6-diamidino-2-phenylindole after immunofluorescence staining. The fluorescence signal was obtained using a fluorescence microscope (DX51, Olympus, Tokyo, Japan).

### Statistical analysis

Comparisons between two groups were made by Student's *t*-test. One-way or two-way ANOVA followed by post hoc Bonferroni test was used when multiple comparisons were made. All data were expressed as mean±S.E.M. A value of *P*<0.05 was considered statistically significant.

## Figures and Tables

**Table 1:** Echocardiographic assessment of left ventricle dimensions and functions in rats

	**Ctrl**	**DM**	**DM Scr-shRNA**	**DM Salusin-*****β*****-shRNA**
LVIDs (mm)	4.66±0.20	5.71±0.18*	5.69±0.20*	5.14±0.17*^†‡^
LVIDd (mm)	8.36±0.30	8.60±0.22	8.35±0.27	8.52±0.18
IVSs (mm)	3.25±0.16	2.56±0.19*	2.51±0.18*	2.53±0.14*
IVSd (mm)	1.91±0.14	1.70±0.13	1.75±0.15	1.73±0.13
LVPWs (mm)	3.20±0.15	2.57±0.09*	2.49±0.13*	2.79±0.14*
LVPWd (mm)	1.96±0.06	1.74±0.08*	1.76±0.05*	1.83±0.07
LVVs (*μ*l)	103±11	165±13*	157±11*	137±9*
LVVd (*μ*l)	383±16	392±21	387±18	398±18
LVM (g)	1.11±0.10	0.95±0.11	0.92±0.08	0.91±0.07
LVM/BW (g/kg)	1.85±0.13	2.30±0.16*	2.26±0.11*	1.88±0.10^†‡^
EF (%)	73.1±2.6	57.9±2.2*	58.1±2.2*	65.6±1.7*^†‡^
FS (%)	44.0±1.3	33.6±1.8*	32.9±1.5*	38.5±1.8*^†‡^

Abbreviations: BW, body weight; EF, ejection fraction; FS, fractional shorting; IVSs and IVSd, interventricular septum at end systole and diastole; LVIDs and LVIDd, left ventricular internal dimensions at end systole and diastole; LVPWs and LVPWd, left ventricular posterior wall dimensions at end systole and diastole; LVVs and LVVd, left ventricular volume at end systole and diastole; LVM, left ventricular mass Values are mean±S.E.M. *P<0.05 *versus* Ctrl; ^†^*P*<0.05 *versus* DM; ^‡^*P*<0.05 *versus* DM/Scr-shRNA. *n*=6
