# Supplementary material for: Salusin-β contributes to oxidative stress and inflammation in diabetic cardiomyopathy
Source: Cell Death Dis. 2017 Mar 23;8(3):e2690–. doi: 10.1038/cddis.2017.106 (PMC5386515; doi:10.1038/cddis.2017.106)
Supplement: Supplementary Materials [file cddis2017106x1.docx]

**Supplementary Materials**

(5 supplementary figures and 2 supplementary tables)

**Salusin-β contributes to oxidative stress and inflammation** **in diabetic cardiomyopathy**

Ming-Xia Zhao^1^, Bing Zhou^1^, Li Ling^1^, Xiao-Qing Xiong^1^, Feng Zhang^1^, Qi Chen^2^, Yue-Hua Li^2^, Yu-Ming Kang^3^, Guo-Qing Zhu^1,2^*****

^1^Key Laboratory of Cardiovascular Disease and Molecular Intervention, Department of Physiology, Nanjing Medical University, Nanjing, Jiangsu 211166, China; ^2^Department of Pathophysiology, Nanjing Medical University, Nanjing, Jiangsu 211166, China; ^3^Department of Physiology and Pathophysiology, Cardiovascular Research Center, Xi'an Jiaotong University School of Medicine, Xi'an 710061, China


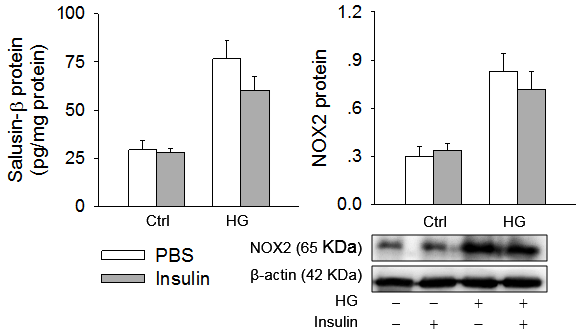


**Supplementary Figure 1** Effects of insulin on salusin-β levels and NOX2 protein expressions in H9c2 cells. H9c2 cells were pretreated with PBS or insulin (100 nM) for 4 hours before HG (33.3 mM) for 24 h. Values are mean±S.E.M. *P<0.05 vs. PBS, ‡P<0.05 vs. Insulin.. n=6.

**
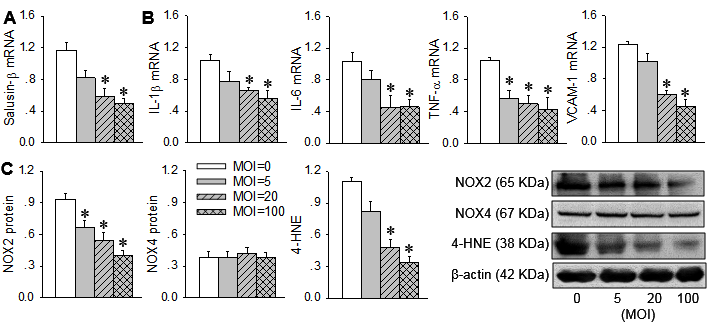
**

**Supplementary Figure 2** Dose effects of Ad-Salusin-shRNA. H9c2 cells were treated with Ad-Salusin-shRNA (0, 5, 20 or 100 MOI) for 24 h. (A) Salusin-β expression. (B) Inflammation. (C) Oxidative stress. Values are mean±S.E.M. *P<0.05 vs. 0. n=6.


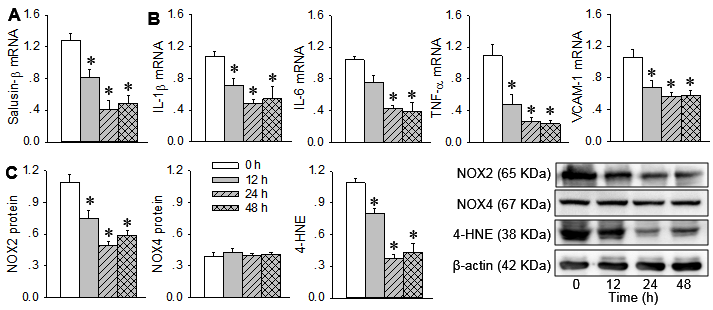


**Supplementary Figure 3** Time effects of salusin-β-shRNA. H9c2 cells were treated with Ad-Salusin-shRNA (100 MOI) for 0, 12, 24 or 48 h. (A) Salusin-β expression. (B) Inflammation. (C) Oxidative stress. Values are mean±S.E.M. *P<0.05 vs. 0 h. n=6.


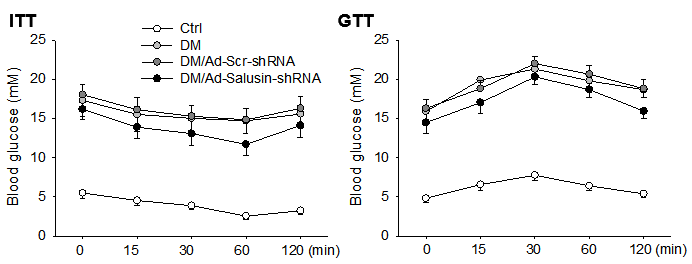


**Supplementary Figure 4** Intravenous injection of adenoviral vectors encoding salusin-β shRNA had no significant on insulin tolerance test (ITT) and Glucose tolerance test (GTT) in diabetic rats (DM). Injection of Ad-Salusin-shRNA (2.0×10^10^ plaque-forming units) or scramble shRNA (Ad-Scr-shRNA) were carried out and repeated in 2 weeks in rats. The measurements were made 4 weeks after the first adenovirus transfer. Values are mean±S.E.M. n=6.


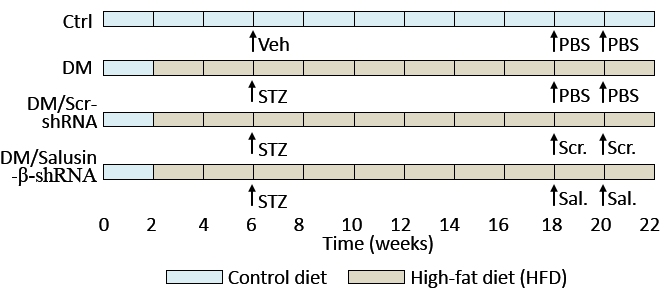


**Supplementary Figure 5** A schematic diagram showing the experimental protocols in control rats (Ctrl) and diabetic rats (DM). Scr., adenoviral vectors encoding scramble shRNA (Scr-shRNA); Sal. adenoviral vectors encoding salusin-β shRNA (salusin-β-shRNA).

**Supplementary Table 1** Sequences of scrambled shRNA and salusin-β-shRNA

| Scrambled shRNA | Sense | 5'-gatccGTTCTCCGAACGTGTCACGTTTCAAGAGAACGTGAC ACGTTCGGAGAACTTTTTTACGCGTg-3' |
| --- | --- | --- |
|  | Antisense | 5'-aattcACGCGTAAAAAAGTTCTCCGAACGTGTCACGTTCTCT TGAAACGTGACACGTTCGGAGAACg-3' |
| Salusin-β-shRNA | Sense | 5'-gatccGCCCTTCTTGGGTTGTGTATGTTCAAGAGACATACAC AACCCAAGAAGGGCTTTTTTa-3' |
|  | Antisense | 5'-agcttAAAAAAGCCCTTCTTGGGTTGTGTATGTCTCTTGAAC ATACACAACCCAAGAAGGGCg-3' |

**Supplementary table 2** Primers for Real-time quantitative PCR analysis in rats

|  | **Forward** | **Reverse** |
| --- | --- | --- |
| **GAPDH** | GTGGAGTCTACTGGCGTCTT | TGCTGACAATCTTGAGGGA |
| **Salusin-β** | TCACTTCTCTCCTATCATCCACTCC | GGCAGCTTGTCCATCTCATCG |
| **NOX2** | TTTCCGATCCTATCAAAGTGCC | GTACACGTGCGTGTGTCTGTTC |
| **IL-1β** | CACCTCTCAAGCAGAGCACAG | GGGTTCCATGGTGAAGTCAAC |
| **IL-6** | AAACCCTAGTTCATATCTTC | CTTAGCCACTCCTTCTGT |
| **TNF-α** | TACTCCCAGGTTCTCTTCAAGG | GGAGGCTGACTTTCTCCTGGTA |
| **VCAM-1** | TACATGTCATGGGACTGTTCCG | CCACAAACCAAGCTATGCATTC |
